# Learning from reproducing computational results: introducing three principles and the *Reproduction Package*

**DOI:** 10.1098/rsta.2020.0069

**Published:** 2021-05-17

**Authors:** M. S. Krafczyk, A. Shi, A. Bhaskar, D. Marinov, V. Stodden

**Affiliations:** University of Illinois at Urbana-Champaign, Urbana, IL, USA

**Keywords:** reproducibility, software testing, code packaging, verification, open code, open data

## Abstract

We carry out efforts to reproduce computational results for seven published articles and identify barriers to computational reproducibility. We then derive three principles to guide the practice and dissemination of reproducible computational research: (i) Provide transparency regarding how computational results are produced; (ii) When writing and releasing research software, aim for ease of (re-)executability; (iii) Make any code upon which the results rely as deterministic as possible. We then exemplify these three principles with 12 specific guidelines for their implementation in practice. We illustrate the three principles of reproducible research with a series of vignettes from our experimental reproducibility work. We define a novel *Reproduction Package*, a formalism that specifies a structured way to share computational research artifacts that implements the guidelines generated from our reproduction efforts to allow others to build, reproduce and extend computational science. We make our reproduction efforts in this paper publicly available as exemplar *Reproduction Packages*.

This article is part of the theme issue ‘Reliability and reproducibility in computational science: implementing verification, validation and uncertainty quantification *in silico*’.

## 1. Introduction

In this article, we investigate current research and dissemination practices to advance understanding and facilitation of the independent reproduction of published results with the same code, data and inputs, and following the same methods. We use the definition of ‘reproducible’ given in the National Academies of Science, Engineering and Medicine 2019 report ‘Reproducibility and Replicability in Science’ [1]:

We define reproducibility to mean computational *reproducibility* – obtaining consistent computational results using the same input data, computational steps, methods, code, and conditions of analysis; and *replicability* to mean obtaining consistent results across studies aimed at answering the same scientific question, each of which has obtained its own data. In short, reproducibility involves the original data and code; replicability involves new data collection and similar methods used by previous studies.

These definitions follow the ‘really reproducible research’ standard introduced by Jon Claerbout in 1994 [2] which has subsequently been developed through a rich history of dedicated research [3–8]. Using this definition of reproducibility means we are concerned with computational aspects of the research, and not explicitly concerned with the scientific correctness of the procedures or results published in the articles. The generation and dissemination of reproducible research is an important, even essential, step for assessing the scientific correctness of computational research, however, since it imbues the computational methods that support scientific claims with transparency and verifiability. Beyond the correctness of scientific claims, poorly engineered code can impede scientific progress by reducing incentives to share research artifacts such as code and data [9], and if they are shared, inhibiting the ease of reproduction and verification of results that rely on them [10,11].

We build on our own previous work that evaluates the potential for reproducing published results in 306 computational physics articles [10]. From this work, we examine seven articles to understand barriers to reproducibility more deeply [12–18]. These seven articles cover a relatively diverse set of topics and use a variety of computational platforms. Our efforts reproducing these articles allow us to define three broad principles to follow when developing reproducible research along with twelve novel guidelines that can be used to apply these principles. We illustrate the importance of the principles and the corresponding guidance through a series of eighteen vignettes taken from our experience investigating the articles. For five of the articles, the authors gave us permission to share their code, and we provide *Reproduction Packages* implementing our guidances. The *Reproduction Package* we define is derived from our empirical research in this article and fills gaps in previous efforts to design and describe the dissemination of linked data, code and results by providing detailed specifications for directory structure, documents and information, as described by our 12 guidelines. Packaging and dissemination for reproducible research has a long history, going back to seminal contributions by Claerbout & Karrenbach [19], Buckheit & Donoho [3], King [4], and Gentleman & Temple Lang’s *Research Compendium* in 2004 [20]. Since then a fruitful line of scholarly contributions on packaging and publishing reproducible research has developed, for example, [21–30].

This article proceeds as follows. After contextualizing our contribution in the Previous Work section, we describe the methodology we used when reproducing each article in the Experimental Design and Methods section. In the Results section, we present the findings from our research reproduction experience. In the Discussion section, we turn our empirical reproduction experiences into principles for the practice and dissemination of reproducible computational research, guidance to follow these principles, and our *Reproduction Package* description. We also include vignettes of our reproducibility experiences that illustrate our principles and guidance. We compare and contrast our approach with existing literature in the Discussion section and propose ways scientists can leverage our principles and guidance. Finally, we leave the reader with some concluding remarks and avenues for future research.

## 2. Previous work

The broad question of reproducibility of published scientific results has long been examined (see, e.g. [31–33]) and our scope in this article is limited to examining computational reproducibility [1], a concept that has emerged over the last several decades. In 2010, the Yale Law School Roundtable on Data and Code Sharing published a manifesto urging more transparency in computationally and data-enabled research and presenting a set of recommendations to enable reproducibility [34]. Peng explains the value of code sharing in 2011 [35]:

[E]very computational experiment has, in theory, a detailed log of every action taken by the computer. Making these computer codes available to others provides a level of detail regarding the analysis that is greater than the analogous noncomputational experimental descriptions printed in journals using a natural language.

In 2016, a set of seven recommendations, the Reproducibility Enhancement Principles (REPs), were published to enable transparency and reproducibility in computational research, and guide researchers and policy makers [36]. Recent work has pointed out public confidence in science can be eroded through a perceived lack of reproducibility of the results [37].

Reproducibility is a complex subject affecting every step in the scientific discovery and dissemination process, including trends [38] and publication bias [39]. In the computational context, when code and data can be extensive, there may simply not be sufficient space in a publication to completely describe the experimental set-up and discovery process. If authors do not or cannot share critical data and computational details, this can prevent reproduction and verification of their work and impede comparisons of any independent re-implementations of the experiment.

With recent efforts showing some high profile works failing to reproduce [40–42], attempts have been made to determine why such works fail to reproduce [43,44], what policies can be taken to decrease reproduction failures [36,45] and whether such policies are effective [11,42,46]. Despite these efforts, scientific results remain challenging to reproduce across many disciplines [11,41,47–58]. We return to these issues in the Discussion section, when we contrast our work with previous and related efforts.

## 3. Experimental design and methods

We build directly on our previous work investigating the state of reproducibility in the field of computational physics [10]. There we examined 306 articles from 10 volumes of the Journal of Computational Physics (JCP), and studied how articles treat their code along with their manuscript text. From the articles and through email requests, we were able to amass code and data for 55 of these 306 articles. Spending a maximum of four hours on each of the 55 articles, we were able to reproduce some aspects of 18 articles but we were *unable* to reproduce any article’s *complete* set of results. In this work, we attempt to reproduce five of those articles for which we have associated artifacts, allocating a maximum of 40 human hours instead of 4, and use this experience to deduce guiding principles to enable computational reproducibility. The 40 h were measured via stopwatch and did not include computational time. In addition to running authors’ experiments, our work included looking up references, emailing and communicating with the authors, and reading necessary background material in order to understand, implement, or fix code we received. Our reproduction team consisted of one postdoc, one graduate student, and one undergraduate student, from computational fields. We estimate human error when starting and stopping the watch to be at most 8 h or 20% in our maximum measured time, based on the non-precise nature of starting and stopping the watch consistently. Our reproductions proceeded through four steps:

(i) **Assess targeted results:** We break each article’s figures and tables into a set of discrete computational experiments to be reproduced.
(ii) **Survey the associated code and data:** We examine any available code and data and attempt to associate these artifacts with the corresponding computational experiments.
(iii) **Modify and write code to run computational experiments:** We ran existing code when possible, adapted code for the computational experiments which were missing code, and wrote new code for examples not covered by the received code.
(iv) **Automate computational experiments and visualizations:** Scripts were then written to run the computational experiments and visualizations to produce figures and tables that either exactly matched, or approximated as closely as we could, those in the original article.

Throughout the reproduction process, we maintained notes in a dedicated **notes.txt** file, available in the open repositories listed in the Data Accessibility statement (we also provide data and code we developed and the original authors’ code when we have permission to do so). We tracked our steps, any roadblocks or difficulties encountered, our solutions to those problems, a running count of what had been reproduced and an estimate of how much of the article we reproduced. This was measured as a percentage of the number of discrete computational experiments identified in step (i). Once we exhausted our allotted time on an article, we restructured the code, scripts, and data into a sharable package described in the following section.

Another group member then used the package to re-execute the code on a different platform and compare the results against the initially obtained values and to check the package for clarity. For each package, we also developed a Dockerfile defining a Docker image with the necessary code to run the computational experiments. Containerization is also well known to assist the reproducibility of computational experiments [59].

Finally, using the results from each article, we produced a set of software tests which can be run on the Travis Continuous Integration (CI)^1^ system. CI is a software engineering practice widely used in industrial and open-source software development [60–62] that works by compiling and executing a set of tests after code changes. A major benefit of such tests for scientific workflows is to highlight points of poor usability, and to know when changes to the code affect scientific findings [63–67].

### (a) Description of chosen articles

We choose five articles from our previous work based on preliminary assessments regarding the potential reproducibility of their results [10]. While reproducing (two of) these articles, we encountered an additional two articles upon whose methods the results depended, and added them to this study. See table 1 for a detailed breakdown.

**Table 1.** Origin of featured articles in this study.

DOI	origin
10.1016/j.jcp.2016.08.012 [12]	JCP Study
10.1016/j.jcp.2016.07.031 [13]	JCP Study
10.1016/j.jcp.2016.10.022 [14]	JCP Study
10.1016/j.jcp.2016.11.009 [15]	JCP Study
10.1016/j.jcp.2016.10.049 [16]	JCP Study
10.1016/j.ces.2015.04.005 [17]	found while trying to reproduce [68]
10.1016/S0377-0427(03)00650-2 [18]	found while trying to reproduce [14]

*Article 1: A fast marching algorithm for the factored eikonal equation.*

Written in the Julia programming language, this article implements a fast marching algorithm to solve the factored eikonal equation [12]. This article was promising because the authors made their code easily available, implemented some software engineering techniques, and their code reproduced one table from the article easily.

*Article 2: A fast accurate approximation method with a multigrid solver for two-dimensional fractional sub-diffusion equation.*

This article defines and implements a new multigrid method for the two-dimensional fractional sub-diffusion equation in the C/C++ programming language [13]. Their method is compared against existing methods to establish its speed and accuracy. We selected this article because the authors gave us code that did not require any external dependencies.

*Article 3: A conservative Fourier pseudo-spectral method for the nonlinear Schrödinger equation.*

Written in Matlab, the authors introduce a new pseudo-spectral method for solving the nonlinear Schrödinger equation [14]. Their method was compared against existing methods to evaluate its speed and accuracy. We selected this article because the authors provided code that did not require external dependencies and produced several rows from the article’s tables. Unfortunately, the authors did not give us permission to share this code.

*Article 4: A structure-preserving scheme for the Kolmogorov–Fokker–Planck equation.*

Written in the Python partial differential equation (PDE) solving framework Fenics [69], this article introduces a new numerical scheme for solving the Kolmogorov–Fokker–Planck equation [15]. We chose this article because the code we received from the authors is clear to read and understand. Unfortunately, the authors did not give us permission to share this code.

*Article 5: Single-node second-order boundary schemes for the lattice Boltzmann method.*

Written in the C/C++ programming language, this article introduces a new way of dealing with boundaries in lattice Boltzmann methods [16]. We chose this article since we had already reproduced portions of it in our previous work.

*Article 6: An extended quadrature-based moment method with lognormal kernel density functions.*

With code written using the C/C++ framework OpenFOAM [70], this article presents a new method for solving population balance equations [17]. We referred to this article to learn the OpenQBMM tool in service of reproducing a different article [68]. We had success reproducing this article, and so it was included.

*Article 7: A modification of Newton’s method for non-differentiable equations.*

Written in Octave, this article presents an implementation of Newton’s method for non-differentiable equations [18]. We encountered this article while attempting to implement a competing method from another article [14]. We included this article as its simplicity led to easy reproduction.

## 4. Results

We present three sets of results in turn: Reproducibility Principles and Guidance, the *Reproduction Package* and Vignettes. The following three principles for enhancing the reproducibility of computational results were derived from our experimental work reproducing the articles studied.

P1. **Provide transparency regarding how computational results are produced.** While a typical article does a good job of conveying the scholarship of the method, namely how the method should work theoretically, a theoretical description of any method alone is not enough to determine what the implementation should be. Methods described in the article can be linked to the fragments of the code implementing the method, and data can be linked to analysis code.
P2. **When writing and releasing research code, aim for ease of (re-)executability.** Transparency alone, however, is not enough. Production of each aspect of the article should also be scripted, and locating the right script to call should be easily done. These scripts should contain any necessary parameters for any computational experiments and be clearly and concisely labelled. Additionally, the specification of resources required is important since other scientists may not have the necessary hardware to complete the computational experiments.
P3. **Make any code upon which the results rely as deterministic as possible.** Re-executing computational experiments should produce exactly the same results as a previous run whenever possible. Software dependencies and versions should be determined, as changes in these dependencies may result in changes in results. If random number generators are used, capture seed information and allow them to be set at the start of a run. Unfortunately, bit-wise reproducible results may not be possible because of inherent non-determinism related to parallelization of an algorithm and associated reduction error, for example, [71]. (This principle is inspired by Rule Ten in [72]).

We present a set of guidelines that put the principles into practice. Like the principles, these results are derived from our experience reproducing the scientific results from the target articles. Recommendations are presented for each guidance.

G1. **Make all artifacts that support published results available, up to legal and ethical barriers.** Authors should make sure all artifacts, such as code and data, they used as part of their computational experiments are available and properly cited whenever possible. Code released by the authors should also come with an appropriate license so users can understand what they can and cannot do with the published code [73].

*Recommendations:*

— Make code open-source and available on widely used open-source software development platforms e.g. GitHub.com or bitbucket.org.
— Use an open-access, long-term storage repository capable of issuing DOIs, such as Zenodo.org, to share datasets.
— Select an open license to allow the greatest flexibility in collaborations. The MIT or BSD (or similar) licenses are acceptable for code sharing; for data sharing, we recommend the CC0 Public Domain Mark; and CC-BY License for text and media as recommended by the Reproducible Research Standard [73].
— If licensing, export control, confidentiality (e.g. HIPAA protection), or excessive size prevents an artifact from being shared, consider producing a synthesized version of data that can be shared. Reporting results for this dataset as well will allow users to verify that the code is working properly even without the primary dataset.

Principles: P1, P2, P3. Source article: [12].

G2. **Connect published scientific claims to the underlying computational steps and data.** Provide a master script that runs all aspects of the computations reported in the publication. Providing this script makes clear to others how the computational experiments are meant to be run to reproduce the findings from the article. Whenever possible, authors should use the same terminology in their code as in their article. For example, if input data is filtered by a pre-processing method, it should be called by the same name in the code as in the article (or should be commented appropriately). Such practice makes clear the connection between methods mentioned in the article and those used in code.

*Recommendations:*

— Endeavour to make parameters used for each experiment easily understood by the user. Provide clear explanations if their purpose is not obvious.
— Ensure no interactivity is needed. Requiring the user to interact with the script requires the user to know aspects of the experiment that may not be obvious.
— Implement the master script in a scripting language. It can be written in shell, Python, Matlab, or another appropriate language, as long as it easy to run.
— For general-purpose software/libraries, include scripts that properly use that software and reproduce results from the article.

Principle: P1. Source article: [68].

G3. **Specify versions and unique persistent identifiers for all artifacts.** Authors should use version control for their work. When they do, they should specify information to uniquely identify the precise code they used when producing the results. When using a version control system, there is usually a commit hash or revision number that uniquely identifies each version. Without version control, identification can be done by specifying the base software version used along with any patches that have been applied.

*Recommendations:*

— Use some form of version control, for example, a tool like Git or Mercurial.
— If version control use is not possible, save different versions of their code explicitly with version numbers. Mention explicitly the version used to produce results for publication.
— In the event that a simulation cannot be re-done after a bug fix, for example, because resources have already been exhausted, then provide multiple commit hashes or unique identifiers that have been used.

Principle: P1. Source article: [17].

G4. **Declare software dependencies and their versions.** Software frameworks are often convenient or necessary to perform scientific computations. Many frameworks depend on other software packages for their functions, but each of these dependencies may evolve over time at different rates. The authors should provide detailed version information for each of these dependencies; such information will make it possible to reconstruct the original software stack used to produce the results.

*Recommendations:*

— Package the code into a Docker [74] image or other container solution such as Vagrant or Singularity [75], which can ‘save’ all necessary dependencies of a computational experiment. Prepare the Docker image using a Dockerfile rather than manually, which captures the knowledge of all dependencies needed to use the computational experiment.
— Use a package management system such as Spack [76] or Conda.^2^ These systems require building all necessary dependencies, and they track the version information of those dependencies as well.
— Use a build system (described in detail later in this article), which enforces an understanding of what the necessary dependencies are for the computational experiments and forces explicit build code to accommodate dependencies.

Principles: P2, P3. Source articles: [15,17,18].

G5. **Refrain from using hard coded parameters in code.** When several computational experiments are conducted by varying one or more parameters, authors should provide a method to define these values at runtime (the time the program is executed). While prior guidance concerning computational scripts is related, it is important to design such scripts to allow for other scientists to change parameters easily and run the experiment again.

*Recommendation:*

— Design computational experiments to either take parameters and arguments from the command line or have them defined in another file, especially when function invocation orderings are important. Documentation and help options are good ways to inform the user of the existing options.
— If parameters are hard coded to achieve compile-time optimization, add compilation of the simulation program to the master script mentioned above. This allows the user to easily configure the hard coded parameters, but also achieve compile-time optimization.

Principles: P1, P2. Source articles: [13,16,17].

G6. **Avoid using absolute or hard-coded filepaths in code.** Absolute file paths such as C:∖Project∖Dataset or /home/user/Dataset are almost certain not to work on a different system from the one where the experiments were initially run. Authors should use file paths defined relative to the current directory, e.g. ./Dataset or ./data/Dataset.

*Recommendations:*

— Start file paths with . or ..
— Look for common giveaways of absolute file paths like a specific username or the name of an external hard drive and eliminate them to create relative file paths.

Principle: P2.

G7. **Provide clear mechanisms to set and report random seed values.** When working with algorithms which rely on random number generators, the authors should implement their script to allow the user to set the initial seed value. Setting the seed value allows the same computational results to be produced on subsequent runs of the program. Furthermore, the authors should report the random seed they used for the results presented in their publication.

*Recommendations:*

— Make sure the seed value can be set and report the seed value used for the article experiment.
— Avoid setting the seed value to changing quantities like the time, or allowing the user to disable such setting.
— Allow the user to set the seed value, for example, by providing an option passable through the command line.

Principle: P3.

G8. **Report expected errors and tolerances with any published result that include any uncertainty from software or computational environments.** Not all results can be made bit-wise reproducible (exactly the same as what was published down to every bit!), resulting in differences between what the author expects and what the code produces (error). In these cases, a tolerable level of error (or tolerance) should be determined based on the application and/or scientific background of the problem. Authors should report that error bound and/or implement a test incorporating the error bound. If a user produces a result different from that published and with no tolerance stated, they may believe they have produced the result incorrectly when they have not.

*Recommendations:*

— Re-run the experiments with slightly different inputs to quantify how the results change. Experiments involving parallelized code may not even need different inputs to observe changes in results as parallelism usually has some non-determinism that can be quantified.
— Provide a script that checks the produced numbers to see whether they are within a tolerance instead of checking that they equal the exact expected values.

Principle: P3. Source articles: [13,18].

G9. **Give implementations for any competing approaches or methods relied upon in the article.** When making performance arguments against competing methods, authors should provide details about how the competing method is implemented and performance tested [77].

*Recommendation:*

— Include the competing code used as part of the artifacts shared. If the authors of the competing code do not give permission to share, point to where the code can be found, including specific version or commit information. Sharing patches to a publicly available software version is important.

Principle: P1. Source articles: [13,14].

G10. **Use build systems for complex software.** When creating complex software, installation procedures can become complex. Even when code consists of a single .cpp file, compiling the code may require linking with additional libraries that may not be clear to users besides the authors. Authors should provide clear instructions for how to install the software.

*Recommendation:*

— Use a build system, such as GNU Make^3^ or CMake^4^ for C/C++ code, to build the code [78].
— Building in a containerized environment can help expose any hidden software dependencies. For example, Python modules may exist in the author’s Python environment, and are not explicitly installed.

Principle: P2. Source articles: [13,16].

G11. **Provide scripts to reproduce visualizations of results.** Authors should provide scripts that produce the figures and tables not produced by hand. Existence of such scripts allows other scientists to see what software is necessary to produce the figures and tables as well as what steps are needed. In our experience, it is frequently assumed that scientists will be able to produce plots as published on their own when the procedure is not clear (such as for a scientist in an adjacent domain). The effort involved in such reproductions without explicit instructions through scripting can be extraordinarily time-consuming or outright impossible. We create this guidance to underscore that it is essential to provide visualization and other scripts as well as those recreating numerical results.

*Recommendation:*

— Provide a clearly identifiable script that creates each figure or table.

Principle: P2. Source articles: [12,14,15,17,79].

G12. **Disclose resource requirements for computational experiments.** Cutting edge research often requires the use of large amounts of computing resources, whether in the form of huge RAM or many computational nodes. As a basic step, authors should report the hardware on which they ran the computational experiments. If possible, including a small test case that can be run by users with conventional hardware is a good step towards not only testing that the code works well, but also allowing others to verify that they can get and run the code correctly before moving to large computational resources.

*Recommendations:*

— Report the hardware on which the experiments were run to obtain the results in the article.
— When running the code, use the time command to measure how long the experiment takes and report this time as a rough estimate for the expected runtime of the experiment.

Principle: P2. Source articles: [12,14,15].

### (a) The *Reproduction Package*: reproducibility and open access

The *Reproduction Package* defines a set of technical standards for the dissemination of reproducible computational research that includes digital artifacts, and extends many previous efforts [21,24,27,80–82]. We first propose a minimal requirement on the directory structure:


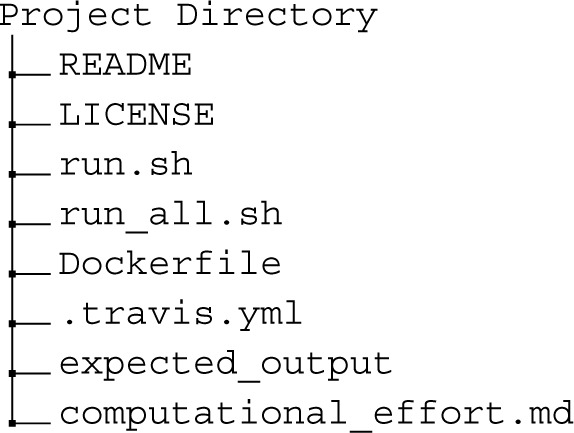


which is defined as follows:

— README: A simple document explaining the reproducibility package and how to use it to reproduce results. It contains at a minimum:
  • Code description
  • Link to the original research article
  • Directions on how to cite this work
  • A description of any software dependencies
  • A description of how to execute the computational experiments from the original research article.
— LICENSE: An essential component is a license telling the user how they are allowed to use the code, following e.g. the Reproducible Research Standard [73].
— run.sh: This script is intended to reproduce the computational experiments in the article that can be run in under 1 h with a reasonably modern desktop computer, with information about expected runtime.^5^
— run_all.sh: This script runs all the computational experiments in the article. In some cases, this script is not needed as experiments are fast enough to be fully included in run.sh.
— Dockerfile: This file helps to make clear how to build the code and any dependencies needed. This file also includes information on running the computational experiments.
— .travis.yml: This script integrates the code with Travis CI. This step shows the reader how to run the experiments.
— expected_output: A directory to store expected output from computational experiments. We recommend only storing finished results that appear in the associated publication such as tables and figures. Other artifacts, such as datasets, may present size limitations for version control systems such as Git. There could be multiple directories containing expected output perhaps categorized by output type such as expected_tables.
— computational_effort.md: This document informs the reader of the computational effort and resources required to execute each experiment. It can warn the reader if some calculations will take a very long time or require boutique environments.

We call this abstracted format for sharing the code and data a *Reproduction Package*. In our experience, the *Reproduction Package* format can be applied to any software and other artifacts packaged for reproducibility. It extends previously established standards and recommendations by including standard ways of storing expected output and reporting expected computational effort [24,27,83]. We also improve on previous methods by increasing the flexibility of the format. By not creating requirements around code and helper script locations, the format can be applied more easily to more situations, increasing the likelihood of adoption. This change is crucial for languages such as Ruby or Julia whose packages expect a specific directory structure. For every article we studied, verification of the results by another team member from the output we produced took less than 5 h of human work. We believe this improvement in time from 40 h shows the value added to the code by the restructuring process induced by the *Reproduction Package*.

In addition to sharing the code and data as a *Reproduction Package* on GitHub, we also include in our packages information specific to our reproduction efforts for the original article, such as the **notes.txt** mentioned previously. We also include a plot of our time spent on reproducing the results for the article and the results yielded by the efforts.

We encourage readers to download our *Reproduction Packages*, run the code and examine how they have been built. Our goal is to provide clear examples showcasing the release of scientific artifacts that enable others to build, run, and verify the results before extending them.

### (b) Vignettes illustrating principles and guidance

**Figure 1.**
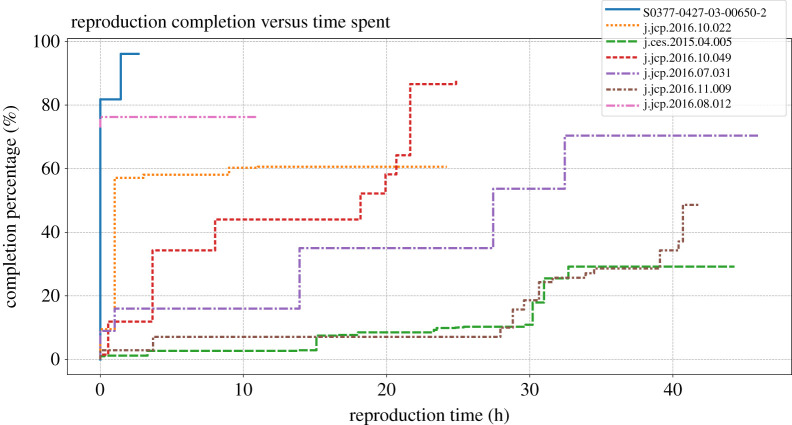
Our efforts to reproduce each article took varying amounts of time (up to 40 h) and yielded varying levels of success. We tracked our progress on each article by noting when significant events occurred and how much of the article we had reproduced at each of these points. Progress is measured by first enumerating the number of computed numbers within tables and plots within figures, referred to as ‘assets’. We rate our completion of each article as the percentage of assets that have been reproduced. Details about which assets were completed when are recorded in each *Reproduction Package* with a file namednotes.txt. We present the completion percentage over time for each article in this study. The solid line is for article [18], the dotted line is for [14], the long dashed line is for [17], the short dashed line is for [16], the long dash dotted line is for [13], the short dash dotted line is for [15] and the long dash double dotted line is for [12]. For each figure, the *x*-axis is time measured in hours; the *y*-axis is the completion measured in percentage. Occasionally, we reached insurmountable roadblocks, terminating our reproduction effort prematurely (before 40 h had been spent). (Online version in colour.)

To illustrate the guidance just presented, we created a set of vignettes from our experience with the seven scientific articles that demonstrate how the problems we encountered can be overcome by following our guidelines. Each illustrative vignette focuses on a single article, though it may apply to more than one. With these condensed stories we aim to show the result of what were dozens of hours of work, as shown in figure 1, and point to gaps encountered by assiduous authors. Occasionally, we reached insurmountable roadblocks, also described in the vignettes.

#### (i) Vignette 1: Missing dataset prevents reproduction

Near the end of their article, the authors perform several computational experiments using a geological model published on a CD with a book [12,84]. We tried to get this book, but the nearest copy was in Barcelona, far from our location, making collecting the book prohibitively time-consuming. Without this model, we could not complete the figures that relied on this data, preventing us from full reproduction of the article even before reaching our 40 h limit. We commend the authors, however, as the details they provided allowed us to reproduce the other results in their work. Guidance: G1.

#### (ii) Vignette 2: Choice of root matters

In [18], we attempted to reproduce several numbers reported in the article. However, our computed numbers were slightly different than those reported. Our inspection found that the differences were due to a choice of root in the solving of a polynomial equation. One part of the procedure required choosing the smallest root of an equation. However, the authors seem to have chosen a slightly larger root than the smallest one, which led to a discrepancy between our results. This experience indicates the need for authors to provide the exact inputs and data used to run their computations. Guidance: G1.

#### (iii) Vignette 3: Finding input by parsing images introduces variance in output

Reproducing results from [17] required extracting moments from a distribution published in a prior work. Initially, we had trouble finding which article contained the distributions but the authors were able to point us to the correct location. The authors themselves stated they had used a plot digitizer program to extract this distribution. We also used a plot digitizer,^6^ and extracted the necessary moments. The results produced from this procedure, however, differed from those published in the article. Unfortunately, the moment inversion algorithm is very sensitive to input differences, especially in the higher-order moments. This means variations in digitization (for example those produced by using a plot digitizer which requires human intervention) can affect the resulting inverted distribution, sometimes significantly, as in our case. Guidance: G1.

#### (iv) Vignette 4: Original version of code unavailable

In one article, figures 12 and 13 seemed to be an application of the moment inversion algorithm mentioned in the previous vignette, this time testing the method with many nodes and an analytic solution [17]. We found that the first three subfigures we reproduced generally matched well with the article. However, beyond these subfigures, the inversion algorithm failed and produced strange results. Additionally, these failures occurred with large numbers of nodes, possibly indicating a numerical instability. Figure 14 references a function *f*(*σ*) for each of four cases. We interpreted this to mean the function from the article’s equation (23), however, this led to rather different results. The lack of clarity regarding this function meant we could not resolve these differences ourselves before our allotted 40 h time limit ran out. The authors communicated readily with us and we learned that they wrote their software with Matlab. Without access to their Matlab code, and without additional time to converse, we were unable to resolve either of these issues. Guidance: G2.

#### (v) Vignette 5: Code provides a check on article correctness

In an article we studied, two numerical examples are presented [17]. These examples are distinguished primarily by changes in two source functions, *f* and *p*, involved in the Laplacian calculation.

From the authors we received code that contains two primary computational experiments: a file named example2.cpp, and a file named example3.cpp (although there is no example 3 in the article). example2.cpp was initially commented out, and further inspection revealed that it did not resemble either examples 1 or 2 from the article. example3.cpp seemed to be a near perfect match for example 2 from the article. However, we noticed some differences between the code and the article. Close inspection revealed a typo in the article’s definition of example 2, however the code in example3.cpp was correct. We demonstrate the error and correction below:

$$\begin{array}{rl} f(x,y,t) & =\frac{\Gamma (4)xyt^{3-\alpha }}{\Gamma (4-\alpha )}-\exp(xy+x^{2}+y^{2})t^{3}\\ & \to \frac{\Gamma (4)xyt^{3-\alpha }}{\Gamma (4-\alpha )}-(x^{2}+y^{2})\exp(xy)t^{3}. \end{array}$$

Once we determined that neither example2.cpp nor example3.cpp contained code directly related to example 1, we went about taking code from the given examples and implementing example 1 ourselves. Changing *f* and *p* functions accordingly yielded correct results for example 1. In total, we matched three computational experiments with the examples from the article: example1.cpp, example2.cpp, and example3.cpp (contains the extra example we received from the authors). Despite the difficulties we had figuring out what code was for which section of the article, the authors’ code was easy to compile and run and thus it was easy to spot and fix this issue. Guidance: G2.

#### (vi) Vignette 6: Inconsistency between analytic computations and equations

In one article we studied, after determining how to run the computational scripts using the correct parameters from the article, we managed to get results that were close but not quite the same as reported [15]. We resorted to a careful inspection of the reported analytic solutions in the article and their derivations to better understand whether the options we were passing to the simulation code were correct. This inspection revealed inconsistencies in the reported analytic solutions. For example, we discovered an error in equation (20) from the article [15]. The denominator should read $\sqrt{1+4t+\frac{4}{3}t^{2}+\frac{4}{3}t^{3}+\frac{4}{3}t^{4}}$ instead of $\sqrt{1+4t+\frac{4}{3}t^{2}+\frac{4}{3}t^{2}+\frac{4}{3}t^{4}}$. This equation is passed to the primary program as an option on the command line. Had the authors provided clear scripts for their experiments, we believe it would have been easier to determine whether they included this mistake in their experiment or whether it is a simple typo only in the paper. Guidance: G2.

#### (vii) Vignette 7: Lack of computational scripts leads to confusion

Although the code given by authors ran without crashing, we could not reproduce the output reported in article [15]. Further inspection of the code, and especially how aspects of the simulation were determined, revealed a possible issue with resolution setting. In the article, the variable ***N*** defines the size of finite elements within the mesh to be such that ***N*** elements fit along the side of the computational domain. The parameter N (from the code) seemingly is also the number of elements along the side of the domain. However, the code uses a library for mesh generation which computes the finite-element size using N as the number of elements along the diameter of a bounding circle, rather than along the side of the domain. This means that to set ***N*** from the article to 128, we need to set N to 177 which is $∼\sqrt{2}N$. After this change, our results matched the article’s more closely. Had the authors included succinct scripts, the link between ***N*** and N would have been clear. Guidance: G2.

#### (viii) Vignette 8: Lack of driver code results forces us to learn a framework

Scientists sometimes release general libraries implementing their methods. For example, ideas from an article we studied were used in the implementation of OpenQBMM,^7^ an extension to OpenFOAM^8^ [17]. Despite test cases inside OpenQBMM explicitly mentioning this article, it was not clear to us how several of the figures were generated. While this general library has code that can perform all or nearly all necessary computations, not all computations were easily exposed and accessible. Extensive work and communication with the authors was necessary to produce several figures and numbers from the article. (The authors used the GitHub platform to great effect for this!) General use libraries are typically developed with a purpose different from reproducibility of published results. For ease of use, developers may try to wrap low-level functionality away from the user, making it hard to reproduce all aspects of the original articles. While use cases may be hard to predict, if authors include test cases from articles that rely on their libraries, missing features or bugs affecting the code output could be more quickly discovered. Guidance: G2.

#### (ix) Vignette 9: Inconsistency between article-described algorithm and code prevents full reproduction of results

Upon close inspection of one article and the code we received, we found their implemented algorithm differed from that described in the article in two ways [13]. First, the multigrid algorithm was named Vcycle_BLTDTDB, and its gross structure did not resemble that described in the article. Second, the smoothing algorithm in the article is named ZLGS, which is different from that mentioned in the code: AXLGS. We carefully inspected this code, but we could not with certainty say that the implemented method is the same as the article-described algorithm. Within our 40 h time limit, we were able to re-implement the multigrid method as described in the article but not the smoother. After these changes, differences between our code and the reported values from the article were now within 1–5% of the reported error value. We also point out that the authors wrote their code in an easy-to-read, logical fashion, making the structure of their algorithm clear through their function calls. This clear structure made re-organizing the code as described above straightforward. Guidances: G2, G3.

#### (x) Vignette 10: Visualization scripts stored in Git history, not explicitly

The authors of one article we studied shared a substantial amount of code for their article through GitHub, but we could not find the code for the figures [12]. Inspecting their commit history revealed past plotting scripts that were removed. We recovered those scripts and were able to develop visualization scripts that reproduced the figures in the article. While our work on these figures would have proceeded more quickly had the visualization scripts been provided in the released code, the authors’ use of version control allowed us to produce the figures. Guidances: G3, G11.

#### (xi) Vignette 11: Fragile scripts require detailed version specifications

Utilizing ParaView’s Python plotting system, we produced several figures from article [15]. Attempting to reproduce these figures on another machine using a slightly different version of ParaView revealed the fragility of these scripts. The Python scripting API implemented in ParaView breaks frequently. Produced scripts are only useful with the version of ParaView which created them unless there is extensive debugging. In our case, the scripts originally developed using ParaView 5.5.2 did not work on either ParaView 5.0.1 (available with Ubuntu 16.04), or ParaView 5.4.1 (available with Ubuntu 18.04). It is important to report the version of the underlying software used (along with the scripts) so results can be reproduced without crashes. Guidance: G4.

#### (xii) Vignette 12: Dependency uncertainty adds to confusion about reproduction failure

Reproducing one article requires the Fenics framework, which contains more than 40 dependencies [15]. We opted to first attempt to use the latest version of Fenics to avoid complications related to building old versions of these dependencies. This attempt required updating several function calls to Fenics that had been updated since the article’s code was written. Ultimately, we ran into a mismatch between our calculated *L*^2^ error and that reported in the article. We used the code that calculates this error as given to us by the author, so the difference does not originate from us changing the code in some way. Consistently across all computational experiments we performed, we did not match the article’s reported error values and we were unable to track down the source of these differences. One possible factor could be differences between versions of not only Fenics but any of its many dependencies.

In order to see whether any of these differences could be attributed to the updated version of Fenics we were using, we built and used Fenics v. 1.5.0, which the author claimed was the version that they used. This was a lengthy process that forced us to devise and apply many specific patches to make certain dependencies build with our more modern compiler. In the end, however, using the older version of Fenics did not resolve the differences we were seeing. While we made an effort to use only ‘period’ versions of packages for these dependencies, it is possible that we did not find the right versions of some of them. The difference in versions may be what is causing the difference in results, but without the definitive list of dependencies and their versions we cannot know for sure. Guidance: G4.

#### (xiii) Vignette 13: Julia deprecation efforts force us to fork modules

During the preparation of this manuscript, several Julia modules deprecated Julia 0.6. Within Julia modules, a file ‘REQUIRES’ details the necessary Julia module dependencies for that module. This deprecation came by changing the line ‘julia 0.6’ to ‘julia 0.7’ within the modules PyPlot.jl (an official module) and jInv.jl ([12]). This change caused Julia to crash with an error complaining about package incompatibility when running the code and data for article [12].

There is a setup script within our code and data package for an article we studied which ensures acceptable versions of various modules are installed before running the rest of the code [12]. We initially believed that specifying older versions of these problem modules (versions which would still work with Julia 0.6) with appropriate Julia code would resolve this issue, however, it did not. Our close inspection of Julia 0.6’s package management source code revealed the latest version of a package is always downloaded and checked for compatibility before the specified version. While this may get fixed in later versions of Julia, we opted to find a way around this problem and preserve our use of Julia 0.6. Further inspection of the Julia source code revealed that a GitHub repository containing a Julia module can be specified. We were thus able to fork these modules and set their master branches to versions which supported Julia 0.6. Once the setup code was adjusted to point to our versions of these modules, the code package worked again. Rather than completely changing Julia, or duplicating the modules we needed, we focused on those modules causing us problems. We cannot therefore guarantee that our code package for Julia will not break again in this manner. Because of the nature of Julia’s package system at version 0.6, a better solution is not apparent at this time. Guidance: G4.

#### (xiv) Vignette 14: Different library versions can lead to different results

For one article, we re-implemented the proposed method using Octave, producing the same results to machine precision [18]. We found that the results could change depending on the version of Octave being used. For our initial experiments, we installed the latest version of Octave available to our platform, namely, v. 4.4.1. However, porting our experiments into Travis CI, we found slightly different numbers for two values (0.00837733 vs. 0.00837735, and 0.41411889 vs. 0.41411902), as well as different output styles. Trial and error revealed a different version of Octave as the culprit. Initially, we used Ubuntu 14.04 for Travis; Octave v. 3.8.1 is available for that version of Ubuntu. Trying Ubuntu 16.04 gave us access to Octave v. 4.0.0; differences in values went away, but the different output styles remained. Building a more sophisticated script to compare output results to expected results allows us to handle the output formatting issue.

In this case, the differences we encountered were fairly small (∼10^−7^). However, more serious software changes can easily lead to much larger differences. Based on this experience, we determined that, when publishing results, it is imperative to share the versions of software used. Without this information, small differences can lead to uncertainty as to whether the results were reproduced properly. Another way to head off this issue is to provide an expected range of error. Had the authors mentioned that we should expect a computational error of 1%, we would not have been worried by the differences we observed. Guidances: G4, G8.

#### (xv) Vignette 15: Hard-coding of parameters leads to confusion and difficulty reproducing experiments

The authors of one article were responsive to our requests for code [16]. We discovered, however, that it was necessary to change hard-coded values to get the reported results for different experiments. Unfortunately, it was difficult to determine which variables in the code had to be changed for which experiments from reading the code. The authors had comments in their code, but the comments were not written in English (we later received translations of these comments from the authors). We eventually had to analyse the equation structure in the code to find where the appropriate changes should be made. In total, it took us several hours to test and determine which variables (in the code) should be changed for each computational experiment. These hours of work would not have been necessary, if the critical variables could be set on the command line.

We found that the use of compiled languages such as C/C++ imply additional care to avoid compile-time constants when they are tied to varying properties of the computational experiment. For example, when performing experiments that vary the grid resolution for some discrete computational method, this resolution should not be coded as a compile-time constant unless there is a compelling performance reason. In most cases, compile-time constants of this type can be avoided, and we demonstrate how to do it with the *Reproduction Package* we produced for one article we studied [16].^9^. Guidance: G5.

#### (xvi) Vignette 16: Irreproducibility of the prior method can render published comparisons impossible

This article sought to show that the author’s new method has better performance and accuracy compared to a traditional method [14]. The authors provided us immediately with an implementation of their method upon request. We quickly verified their reported performance for their method. However, we did not get an implementation of the competing method. We attempted to re-implement the competing method after referencing materials on how the method should be implemented [18,85]. Unfortunately, our implementation efforts did not match those reported in the article. The error our implementation achieved was several orders of magnitude worse than that reported in the article. We resorted to asking the authors for the implementation of the competing method as well, but we did not get any response. Without this implementation, we were unable to verify the reported accuracy and performance gains of this new method (although we could verify the direction of the gains). We also ran into this problem with another article we studied [13]. In that case, we were able to produce an implementation of the competing method. Guidance: G9.

#### (xvii) Vignette 17: Reproducibility issues for visualizations using ParaView

**Figure 2.**
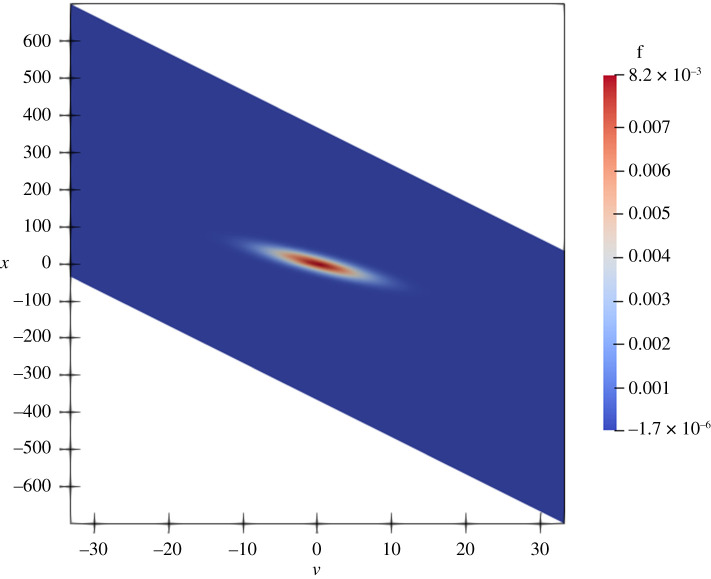
Reproduction of figure 5c from article [15] after the transformation with conspicuous empty spaces. (Online version in colour.)

**Figure 3.**
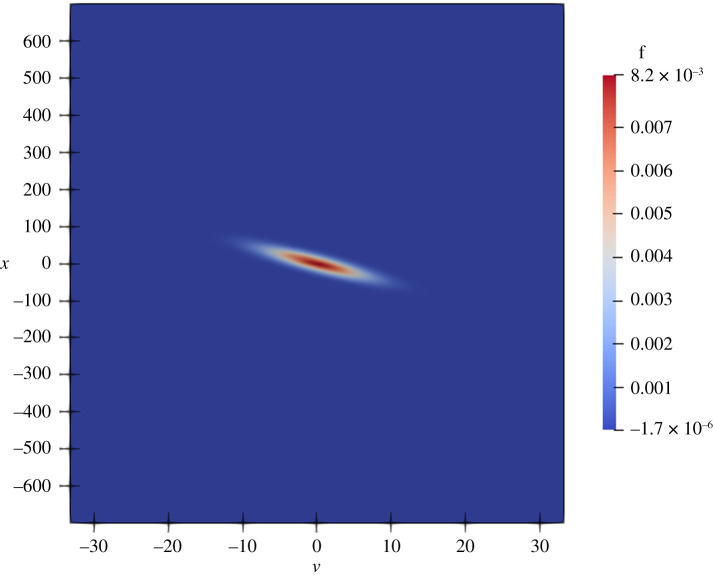
Reproduction of figure 5c from article [15] after the transformation and with conspicuous empty spaces removed. (Online version in colour.)

In an article, several two-dimensional density plots are presented [15]. However, the software used to produce those figures is not described. The code that the authors provided to us did not include any scripts for creating the plots either. Instead, while reading documentation for Fenics, we found a likely candidate to be the ParaView plotting suite. Utilizing ParaView’s Python scripting interface, we could produce figures *similar* to those from the article and store Python scripts to recreate them. We achieved success with all such figures, except figures 5*c*, 5*d*, and 7*c*. For each of these figures, we needed to perform a variable transformation. ParaView supports variable transformations, but points in the new domain mapping to values not in the old domain are not interpolated with ‘0’ values, resulting in strange stretched rectangular plots with large empty spaces not present in the article. By plotting the original version of the plot underneath the transformed version, this problem was avoided. We demonstrate this effect in figures 2 and 3. Presumably, the authors had to perform similar actions with their visualization scripts, and had we received such scripts, we could have performed this step more easily. Guidance: G11.

#### (xviii) Vignette 18: RAM shortage prevents completion of table data computation

An article presents a table of the proposed method’s accuracy performance as computational grid resolution increases [15]. Unfortunately, we could not reproduce the last row of the table, which required more memory than available on the system we were using (32 GB RAM, Arch Linux, i7-6900K). The authors did not report the amount of memory they used for their results. We also encountered this issue in another article we studied [12], and in previously mentioned prior work [86,87]. Guidance: G12.

## 5. Discussion

We begin our discussion by situating these results within previous work. Many articles have put forward recommendations for publishing computational research and scientific software, and we present our recommendations as an extension of these results. We then discuss the software *Reproduction Packages* we are releasing with this article. We next discuss incentives for researchers and how they may help or hinder the adoption of the guidelines we motivate here. We then consider how the structure of our study can potentially limit the scope to which the guidelines are applicable. Finally, we discuss our use of Travis CI in the scientific research context.

### (a) Relationship to prior work

We build on prior work in infrastructure/tools and also in policy/practice recommendations. New tools and standards can help authors make their work more reproducible. Our approach endeavours to complement and extend the existing body of work, including the generation of and reliance on a broader set of empirically derived findings regarding current practices in computational research to derive principles, guidance, and the technical specifications developed for the *Reproduction Package*.

In the infrastructure and software tool space, there are myriad different approaches that can help increase reproducibility. Containerization technology like Docker [74] and Singularity [75] allow the scientists to bundle operating system components, software dependencies, and scripts together into a convenient package which can be run on most modern computing platforms. Systems like ReproZip [88] make this even easier by simplifying the containerization phase for some types of scientific workflows. Package management systems such as Conda^10^ and Spack [76] manage nearly the entire dependency stack of a given application. These types of programmes are capable of building all necessary software in a managed way. Workflow management systems can capture detailed experiment provenance information for reproducibility and allow scientists to define the workflow of their computational experiments more robustly [89–96]. How to execute computational experiments is made clearer when using such systems. Literate programming systems such as Jupyter [97] allow the authors to weave both narrative and code together, alleviating problems associated with defining computational experiments and linking code to theoretical descriptions. More integrated approaches such as RECAST [98] take advantage of the science of a specific field to allow published analyses to be more easily reproduced. Efforts like Whole Tale [99] aim to create an integrated environment where whole scientific workflows can be written, stored, run, and reproduced, with many components of enabling reproducibility automated.

Discovery and dissemination specifications from efforts such as Popper [24], Big Data Bags [82] and the Whole Tale project’s Tale specification [27], delineate guidance and tools to help scientists conform to more reproducible research practices. These efforts build on reproducibility packages aimed at releasing artifacts associated with reproducible scientific results and developed as early as 1992 [19,21,22,25,26,28,100]. In this work, we provide a novel technical specification for a *Reproduction Package*. This extends previous efforts in two ways. The *Reproduction Package* endeavours to provide concrete guidance on publication details important for reproducibility. Although groundbreaking at the time, previous efforts such as [100,101] provided abstract notions rather than technical specifications for code and data sharing for reproducibility. Through our novel specification we extend the guidance provided by earlier efforts, such as Whole Tale’s ‘Tale’. By building on ideas from [24,102], the *Reproduction Package* integrates important guidance on the directory structure for the research code within the container and computational environment that enables reproducibility and eases discovery pipeline comparisons with other research efforts.

Workshops such as the ICERM Workshop on Reproducibility in Computational and Experimental Mathematics [103,104], WSSSPE [105], P-RECS (Practical Reproducible Evaluation of Systems) or convened committees [106], bring together stakeholders around reproducibility issues. We extend these discussions in the current work, as well as discussions in the published literature [28,72,107–111]. Like previous work [72,107], we provide guidelines, and we also contribute an explicit framing with the three fundamental principles we present. For example, Rule Ten from [72] indicates that identical results should be produced when given identical inputs, and we have extended this to a general principle (our third) and introduced an allowance for differences arising from inherent non-determinism, for instance, computational non-determinism such as scheduling, concurrency, parallelism, or hardware differences among reproducibility runs. Sandve *et al.*’s Rule Six (‘For Analyses That Include Randomness, Note Underlying Random Seeds’) [107] is extended in our Guidance G7 ‘Provide clear mechanisms to set and report random seed values’ even though our articles did not use random seeds since this is an important guidance and another application of scripting in the service of reproducibility. In addition, we note that avoiding interactivity in general is desirable for reproducibility and make this point in Guidance G11 with reference to scripting analysis and visualization steps. We do not make any specific recommendations in general about what tools authors should or should not use in their own work, other than to say that the computational aspects should be open. Software and tools can change rapidly, and this work seeks to contribute principled guidance that can remain relevant beyond any specific set of tools or infrastructure.

### (b) Incentives regarding the principles and guidance

Following our recommended guidance has costs, so we discuss the incentives for the authors to follow these guidelines. On average it took us about 35 h per article to reproduce the findings to some reasonable degree. This amount of time may seem daunting for authors to spend on work which ostensibly appears to be completed, however we believe that our time estimate over-approximates what the authors themselves may experience when trying to enable reproducibility as their research progresses; our estimate is closer to what they might encounter if they attempted to reuse their work, say, a year in the future. Moreover, our team did not always have the domain knowledge or experience with the workflow of the code, unlike the authors, which affected the time it took us to reproduce the findings. As has been noted elsewhere, authors may forget important details of the computational workflow if not explicitly recorded at the time, what part of their code does what, what input parameters were used or how they were estimated, and in what order each aspect of their code must be executed [6,112].

We believe that if the principles and guidance outlined in this article are followed from the *start* of a project several gains can be made. Firstly, the accumulated time spent on these activities will be less than 35 h. Secondly, future reuse of the work will be significantly easier which has the effect of lowering barriers to collaboration and building on previous work. Gains from these factors can make new student onboarding and collaboration with other groups more efficient, as well as imparting greater confidence that the results are computationally correct.

### (c) Implementation of CI tests

As discussed previously, for each of the seven articles, we constructed tests to be run on Travis CI, a service for continuous integration (CI) available at https://travis-ci.org. We constructed these tests based on our attempts at reproducing the results from the articles. In general, we found it very easy to implement tests which involved simply building and running the code successfully on Travis. We implemented tests that compared the results of the computational experiments against previously computed known answers, which took us 2–4 h per article. In fact, most of the time used for these articles consisted of writing an appropriate tool to parse output from these computational tests and then compare it to the known results, the problem sometimes called ‘test oracles’ [113]. The varied nature of the output of each individual test made it impossible to write a general-purpose solution, so each test required a customized solution. With these CI tests, a researcher looking to use these code bases will be able to execute the tests on their own machines and know that they have the correct code performing the correct calculations to a specified level of accuracy. Where and how that accuracy level may be *precisely* set is a question for future work.

Another issue that can arise with implementing CI tests is that researchers may write some *flaky tests*, which are tests that can non-deterministically pass or fail when run on the same version of code [114]. In general, flaky tests can mislead developers during software development: a flaky test that fails after a code change may not indicate a bug in the change, because the test could have failed even before the change. Prior work identified a number of reasons why tests are flaky in traditional software, e.g. concurrency, floating-point computations, or randomness [114]. While we did not encounter flaky tests when we implemented our own CI tests for the cases in this work, those common reasons can show up in code that researchers write for their experiments, easily leading to flaky tests. Thus, flaky tests can present challenges for reproducibility in the research code. Prior work in the software engineering domain proposed techniques to detect and fix flaky tests [115,116]; implementing such techniques to automatically detect and fix flaky tests in research code is a question for future work.

### (d) Limitations of our work

Our work focuses on articles published in the Journal of Computational Physics. While we believe our guidelines can generalize well to other fields of study, it is possible specific projects may modify the guidance and recommendations. We are confident the three principles can provide overarching direction and the *Reproduction Package* can be widely implemented successfully.

Since we interacted with authors and attempted to implement missing features or methods ourselves, our work describes only what is possible given 40 h of human time and solid motivation to reproduce the results. It’s possible that different results would be achieved if the researcher reproducing the results was more skilled in the given field than us, or didn’t have time to implement anything themselves, or if the authors were more or less responsive to requests for comment. We endeavoured to provide a clear exposition of how much effort is needed to gain at least some amount of reproducibility under specific circumstances.

As mentioned in the Methods section, our time measurement was subject to human error. Our experience indicates that more results are unlikely to be reproduced in just a few additional hours of work after 40. Further compounding uncertainty here is the fact that over time we gained skill in reproducing these articles. It is possible that an hour of time on the last article we reproduced was more productive than an hour of time spent on the first article.

## 6. Conclusion

In our experience, the articles and approaches studied in this work showcase widespread issues with scientific code sharing throughout the scientific community. As discussed in the Incentives section, the routine production of fully reproducible published results is not common practice. From our empirical investigations, we contribute three broad principles for reproducible computational research: provide transparency regarding how computational results are produced; when writing and releasing research code, aim for ease of (re-)executability; and make any code upon which the results rely as deterministic as possible. We present 12 guidelines that implement the principles, 18 vignettes demonstrating how these guidelines were derived, and several openly available code packages as exemplars of these recommendations and the *Reproduction Package*.

While we had some difficulty reproducing work published by our initial five target articles [12–16], and then the two related articles [17,18], in the end we were able to regenerate numerically identical results to some of those in the articles, and visually similar figures. Our success was in no small part due to the high-quality code and interest we received from the *authors* of the original five papers.

Through our experiences reproducing these articles, we extend previous efforts to elucidate principles to enable the research community to design and release better research software. Principles are necessarily broad, and we accordingly produce more focused guidance for implementation. Supporting and illustrating our choices for our set of guidelines, we also present a series of vignettes showcasing our experiences with reproductions. The guidelines allow us to develop a technical specification called the *Reproduction Package* for the dissemination of research code, and we published each of the seven reproductions we carried out in this work as a *Reproduction Package*. Improving software quality is known to reduce costs and increase productivity in the software industry [117,118], and we believe following the technical specifications given by the *Reproduction Package* can strengthen the scientific record and accelerate the rate at which new research can be completed, verified, and extended in the research community.

We believe our findings generalize to computationally- and data-enabled research, since the articles we studied represent different computational problems, needs and approaches. Although they do not represent every approach, consider an article which details an effort to reproduce work in computational biology [119] where the authors highlight many of the same issues we encountered.

With an improved understanding of problems with computational reproducibility in a broader set of domains, patterns in code and scripts emerge that can make previous work easier and faster to reproduce. Our goal in proposing clear guidance for how to set up, write and share code and scripts such as the run.sh script is to facilitate general solutions to frequently encountered issues. For example, what assumptions should be made when creating these scripts, such as the hardware or computational environment, how the scripts should be formatted, and what level of documentation should be available within the scripts. Guidelines of this type, which we have endeavoured to provide, can create consistency and reproducibility in computational experiments across the community.

Publishers could provide a template for the master run script run.sh and check the internal structure of software packages that the authors submit to be published alongside their article, thus extending existing standards [7]. While many journals have requirements for data compliance [120], scientists may not adhere to such standards [121]. How publishers should reward and incentivize researchers to comply with standards for reproducibility deserves more study in the future.

Finally, improved use of software testing in the scientific context is an area with increasing interest [63]. As an example, in one article we studied [17], several figures utilize a moment inversion method. A diversity of tests would stress the algorithm and form a potential basis for automated testing of the underlying method. Further research focused on finding out what elements form appropriate tests of scientific code is needed. Ensuring published results are reproducible remains a challenging [119,122] and fruitful area for future research.

## Footnotes

1 https://travis-ci.org.

2 https://conda.io.

3 https://www.gnu.org/software/make/.

4 https://cmake.org.

5 All articles in this study were tested on a desktop computer with an i7-6900K CPU, 32GB of RAM, and running the Arch Linux operating system.

6 https://automeris.io/WebPlotDigitizer/.

7 https://github.com/OpenQBMM/OpenQBMM.

8 https://openfoam.org/.

9 
https://github.com/ReproducibilityInPublishing/j.jcp.2016.10.049

10 https://conda.io.

## Data accessibility

Code and data from the following articles are available at the following URLs:

— [17] is available at https://github.com/ReproducibilityInPublishing/j.ces.2015.04.005.
— [13] is available at https://github.com/ReproducibilityInPublishing/j.jcp.2016.07.031.
— [12] is available at https://github.com/ReproducibilityInPublishing/j.jcp.2016.08.012.
— [16] is available at https://github.com/ReproducibilityInPublishing/j.jcp.2016.10.049.
— [18] is available at https://github.com/ReproducibilityInPublishing/10.1016_S0377-0427-03-00650-2.
